# Supplementary material for: Benchmarking Observational Analyses Before Using Them to Address Questions Trials Do Not Answer: An Application to Coronary Thrombus Aspiration
Source: Am J Epidemiol. 2022 May 31;191(9):1652–65. doi: 10.1093/aje/kwac098 (PMC9437817; doi:10.1093/aje/kwac098)

Web Material

Title: Benchmarking observational analyses before using them to address questions trials do not answer: an application to coronary thrombus aspiration

Anthony A. Matthews, Issa J. Dahabreh, Ole Fröbert, Bertil Lindahl, Stefan James, Maria Feychting, Tomas Jernberg, Anita Berglund, and Miguel A. Hernán

Contents

Pg 3: Web Appendix 1 – Standardization model specification

Pg 4: Web Table 1 – Baseline characteristics of the randomized patients enrolled in TASTE, according to treatment group

Pg 5: Web Table 2 - Covariate definitions and model specification in the target trial

Pg 7: Web Table 3 - Outcome definitions in the target trial

Pg 8: Web Table 4 - Baseline characteristics of eligible individuals from an observational emulation of a target trial of thrombus aspiration vs. no thrombus aspiration, SWEDEHEART registry, 2007-2016, with unweighted and inverse probability weighted standardized mean differences

Pg 10: Web Table 5 - Baseline characteristics of eligible individuals for an observational emulation of a target trial of thrombus aspiration vs. no thrombus aspiration, SWEDEHEART registry, stratified into 2007-2010 & 2013-2016

Pg 13: Web Table 6 - Estimated 1-year risk, risk difference, and risk ratios from an observational emulation of a target trial of thrombus aspiration vs. no thrombus aspiration, SWEDEHEART registry, stratified into 2007-2010 & 2013-2016

Pg 14: Web Table 7 - Sensitivity analysis - Estimated 1-year risk, risk difference, and risk ratios from an observational emulation of a target trial of thrombus aspiration vs. no thrombus aspiration, SWEDEHEART register, 2007-2016, without application of the 50% minimum stenosis eligibility criterion

Pg 15: Web Table 8 - Sensitivity analysis - Estimated 1-year risk, risk difference, and risk ratios from an observational emulation of a target trial of thrombus aspiration vs. no thrombus aspiration, SWEDEHEART register, 2007-2016, adjusted for age and sex

Pg 16: Web Table 9 - Sensitivity analysis - Difference in risk differences between age and sex adjusted, and fully adjusted models from an observational emulation of a target trial of thrombus aspiration vs. no thrombus aspiration, SWEDEHEART register, 2007-2016

Pg 17: Web Table 10 - Sensitivity analysis - Estimated 1-year risk, risk difference, and risk ratios from an observational emulation of a target trial of thrombus aspiration vs. no thrombus aspiration, SWEDEHEART register, 2007-2016, with individuals censored at death

Pg 18: Web Table 11 - Sensitivity analysis - Estimated 1-year risk, risk difference, and risk ratios from an observational emulation of a target trial of thrombus aspiration vs. no thrombus aspiration, SWEDEHEART register, 2007-2016, with a complete case analysis

Pg 19: Web Table 12 - Sensitivity analysis - Estimated 1-year risk, risk difference, and risk ratios from an observational emulation of a target trial of thrombus aspiration vs. no thrombus aspiration, SWEDEHEART register, 2007-2016, with alternative myocardial infarction definition

Pg 20: Web Table 13 - Sensitivity analysis - Estimated 1-year risk, risk difference, and risk ratios from an observational emulation of a target trial of thrombus aspiration vs. no thrombus aspiration, SWEDEHEART register, 2013-2016, additionally adjusted for Killip class

Pg 21: Web Table 14 - Sensitivity analysis - Estimated 1-year risk, risk difference, and risk ratios from an observational emulation of a target trial of thrombus aspiration vs. no thrombus aspiration, SWEDEHEART register, 2013-2016, additionally adjusted for time period (before or after TASTE)

Pg 22: Web Table 15 - Sensitivity analysis - Estimated 1-year risks from an observational emulation of a target trial for thrombus aspiration and no thrombus aspiration, SWEDEHEART register, 2013-2016, when data are stratified by exposure ad outcome models are fit in each arm

Pg 23: Web Table 16 - Sensitivity analysis - Estimated 1-year risk, risk difference, and risk ratios from an observational emulation of a target trial of thrombus aspiration vs. no thrombus aspiration, SWEDEHEART register, 2013-2016, adjusted for baseline confounding using inverse probability weighting

Pg 24: Web Figure 1 - Average 1-year hazard ratio from TASTE and estimated 1-year risk ratios from an observational emulation of a target trial of thrombus aspiration vs. no thrombus aspiration, SWEDEHEART registry, stratified into 2007-2010 & 2013-2016

Web Appendix 1 - Standardization model specification

In the target trial the intention to treat effect is the effect of being assigned to thrombus aspiration followed by percutaneous coronary intervention versus percutaneous coronary intervention alone on the risk of death, myocardial infarction, or stent thrombosis. Estimating its observational analog requires adjustment for baseline confounders, which we achieved through standardization. First, we fit the pooled logistic regression model:

$$logit\left(Pr\left[Y_{m+1} = 0 \mid A, L, Y_m = 0 \right] \right) = \beta_0 + \beta_1 m + \beta_2 m^2 + \beta_3 A + \beta_4 Am + \beta_5 Am^2 + \beta_6^T L$$

where Y_{m+1} is the indicator for the outcome of interest at time $m+1$ (observed among individuals who did not experience the outcome in the previous period, m, such that $Y_m = 0$); A is the indicator for treatment; and L is a vector of potential confounders at baseline.

We then used the predicted probabilities from these models, to estimate period-specific cumulative probabilities of being event-free for each individual, i, under each treatment strategy conditional on the individual's baseline confounders at, L_i. For individual i and period k, we estimated the survival probability under treatment a using the following formula:

$$\widehat{OM_{i,k}^a} = \prod_{m=1}^{k} \left\{ 1 - expit(\hat{\beta}_0 + \hat{\beta}_1 m + \hat{\beta}_2 m^2 + \hat{\beta}_3 a + \hat{\beta}_4 am + \hat{\beta}_5 am^2 + \hat{\beta}_6^T L_i) \right\}.$$

We then standardized the survival probabilities at each time point to the empirical distribution of the baseline confounders:

$$\widehat{OM_k^a} = \frac{1}{n} \sum_{i=1}^{n} \widehat{OM_{i,k}^a},$$

where n is the number of individuals in the study sample. The risk at time k, can then be estimated by taking one minus $\widehat{OM_k^a}$; risk differences and risk ratios can be calculated using the estimated risks (under each treatment). Finally, we used nonparametric bootstrapping with 200 samples to construct 95% confidence intervals.

Web Table 1 – Baseline characteristics of the randomized patients enrolled in TASTE, according to treatment group

Characteristic	No thrombus aspiration (N=3623)	Thrombus aspiration (N=3621)
Mean age (yr) ± SD	65.9 ± 11.7	65.5 ± 11.5
Male (%)	2703 (74.6)	2721 (75.1)
Diabetes (%)	453 (12.5)	448 (12.4)
Current smoking (%)	1173 (32.4)	1083 (29.9)
Previous myocardial infarction (%)	440 (12.1)	402 (11.1)
Previous PCI (%)	362 (10.0)	337 (9.3)
Previous CABG (%)	74 (2.0)	70 (1.9)
Fibrinolysis before PCI (%)	69 (1.9)	69 (1.9)
Procedure related medication (%)		
Acetylsalicylic acid	3542 (97.8)	3546 (97.9)
Clopidogrel or ticlopidine	2395 (66.1)	2384 (65.8)
Ticagrelor	1015 (28.0)	1050 (29.0)
Prasugrel	538 (14.8)	562 (15.5)
Heparin	3074 (84.8)	3063 (84.6)
Low molecular-weight heparin	142 (3.9)	147 (4.1)
Bivalirudin	2835 (78.3)	2874 (79.4)
Glycoprotein IIb/IIIa inhibitor	630 (17.4)	558 (15.4)
Time from diagnostic ECG to PCI (min)		
Median	66	67
25th-75th percentile	47-93	48-95
Killip class ≥ 2 (%)	183 (5.1)	198 (5.5)
Radial-artery approach (%)	2415 (66.7)	2394 (66.1)
Type of disease (%)		
One-vessel	1940 (53.5)	1946 (53.7)
Two-vessel	1072 (29.6)	1010 (27.9)
Three-vessel	498 (13.7)	558 (15.4)
Left main coronary artery disease	105 (2.9)	98 (2.7)
Data not available	8 (0.2)	9 (0.2)
TIMI flow grade 0 or 1 (%)	2811 (77.6)	2821 (77.9)
Thrombus grade (%)		
G0	543 (15.0)	490 (13.5)
G1	809 (22.3)	733 (20.2)
G2	329 (9.1)	341 (9.4)
G3	818 (22.6)	887 (24.5)
G4	863 (23.8)	903 (24.9)
G5	215 (5.9)	235 (6.5)
Unknown	46 (1.2)	32 (0.9)

SD = standard deviation; PCI = percutaneous coronary intervention; CABG = coronary artery bypass graft; ECG = electrocardiogram; min = minutes; TIMI = thrombolysis in myocardial infarction

Web Table 2 - Covariate definitions and model specification in the target trial

Covariate	Register used	Definition	Form	Categories
**Age**	SCAAR		Linear, quadratic	N/A
**Gender**	SCAAR		Indicator	Male/female
**PCI Centre**	SCAAR	Borås, Danderyd, Eskilstuna, Falun, Gävle, Halmstad, Helsingborg, Huddinge, Jönköping, Kalmar, Karlskrona, Karlstad, Karolinska Solna, Kristianstad, Linköping, Lund, Malmö, Sahlgrenska, Skövde, St Görans, Sunderbyn, Sundsvall, SÖS, Trollhättan, Umeå, Uppsala, Västerås, Örebro, Östersund, Östra sjukhuset	30 categories	As in definition
**Stenosis class**	SCAAR	1 - A<br>2 - B1<br>3 - B2<br>4 - C<br>5 - B1 Bifurcation<br>6 - B2 Bifurcation<br>7 - C Bifurcation<br>8 - Other<br>9 - Missing	9 categories	1, 2, 3, 4, 5, 6, 7, 8, missing
**Proportion stenosis**	SCAAR	Proportion stenosis in lesion with maximum stenosis filling<br>1 - 50-69%<br>2- 70-89%<br>3 - 90-99%<br>4 - 100%	4 categories	1, 2, 3, 4
**Angiography finding**	SCAAR	0 - normal<br>1 - 1 vessel + no left main<br>2 - 2 vessels + no left main<br>3 - 3 vessels + no left main<br>4 - left main	5 categories	0, 1, 2, 3, 4
**Body mass index**	SCAAR	Weight/Height^2<br><br>In main analysis missing imputed with median. Any individuals with BMI above 50 set to 50, and individuals with BMI of below 10 set to 10	Linear, quadratic	N/A
**Smoking**	SCAAR	0 - never<br>1 - ex-smoker (> 1month)<br>2 - current<br>9 - missing	4 categories	0, 1, 2, missing
**Diabetes**	SCAAR	If missing assumed no	Indicator	Yes/No
**Prior myocardial infarction**	SCAAR	If missing assumed no	Indicator	Yes/No
**Prior percutaneous coronary intervention**	SCAAR	If missing assumed no	Indicator	Yes/No
**Prior coronary artery bypass grafting**	SCAAR	If missing assumed no	Indicator	Yes/No
**Prior treatment for hypertension**	SCAAR	If missing assumed no	Indicator	Yes/No

Covariate	Register used	Definition	Form	Categories
**Prior lipid lowering treatment**	SCAAR	If missing assumed no	Indicator	Yes/No
**Thrombolysis**	SCAAR	Before or under PCI, if missing assumed no	Indicator	Yes/No
**Warfarin**	SCAAR	Before or under PCI, if missing assumed no	Indicator	Yes/No
**Aspirin**	SCAAR	Before or under PCI, if missing assumed no	Indicator	Yes/No
**Clopidogrel/ticlopidine**	SCAAR	Before or under PCI, if missing assumed no	Indicator	Yes/No
**Prasugrel**	SCAAR	Before or under PCI, if missing assumed no	Indicator	Yes/No
**Heparin**	SCAAR	Before or under PCI, if missing assumed no	Indicator	Yes/No
**Low molecular weight heparin**	SCAAR	Before or under PCI, if missing assumed no	Indicator	Yes/No
**Bivalirudin**	SCAAR	Before or under PCI, if missing assumed no	Indicator	Yes/No
**GpIIb inhibitors**	SCAAR	Before or under PCI, if missing assumed no	Indicator	Yes/No
**Heart rate**	RIKSHIA	Some missing as captured from RIKSHIA and not all individuals have related RIKSHIA record.<br><br>In main analysis missing imputed with median	Linear, quadratic	N/A
**Systolic blood pressure**	RIKSHIA	Some missing as captured from RIKSHIA and not all individuals have related RIKSHIA record.<br><br>In main analysis missing imputed with median	Linear, quadratic	N/A
**Diastolic blood pressure**	RIKSHIA	Some missing as captured from RIKSHIA and not all individuals have related RIKSHIA record.<br><br>In main analysis missing imputed with median	Linear, quadratic	N/A

SCAAR - Swedish Coronary Angiography and Angioplasty Registry; RIKSHIA - Registry of Information and Knowledge about Swedish Heart Intensive care Admissions; BMI = body mass index; PCI = percutaneous coronary intervention; GpIIb inhibitors = glycoprotein IIb/IIIa inhibitor

Web Table 3 - Outcome definitions in the target trial

TASTE trial	Target trial definition
**Death from any cause**	1. SCAAR<br>The SCAAR register was used to identify date of death.<br><br>2. Cause of death register<br>If there was no death record in the SCAAR register, but a record of death in the cause of death registry, then date of death was taken from cause of death register.
**Myocardial infarction**	RIKSHIA<br>a. Between PCI and discharge date<br>If there was a record of reinfacrction related to the individuals record for intial myocardial infarction nd resuts percutaneous coronary intervention, then the date of new myocardial infarction was considered as the date of discharge. The actual date of reinfarction is not rcorded in SWEDEHEART, but the median time between original admission and discharge is 3 days.<br><br>b. After inital discharge date<br>After the individual for the intial period of care following percutaneous coronary intervetion through until the end of follow up, all myocardial infarction records in the RIKSHIA register were used (ICD 10 codes I21/I22).

* SCARR - Swedish Coronary Angiography and Angioplasty Registry
** RIKSHIA - Registry of Information and Knowledge about Swedish Heart Intensive care Admissions

Web Table 4 - Baseline characteristics of eligible individuals from an observational emulation of a target trial of thrombus aspiration vs. no thrombus aspiration, SWEDEHEART registry, 2007-2016, with unweighted and inverse probability weighted standardized mean differences

	Thrombus aspiration	No thrombus aspiration	unweighted SMD	IP weighted SMD
**n**	3462	14760		
**Age (yrs) (median [IQR])**	66.0 [57.0, 74.0]	68.0 [60.0, 77.0]	0.190	0.043
**Female (%)**	887 (25.6)	4422 (30.0)	0.097	0.023
**Hospital (%)**			0.649	0.128
Borås	15 (0.4)	143 (1.0)		
Danderyd	134 (3.9)	363 (2.5)		
Eskilstuna	67 (1.9)	398 (2.7)		
Falun	211 (6.1)	638 (4.3)		
Gävle	333 (9.6)	506 (3.4)		
Halmstad	13 (0.4)	267 (1.8)		
Helsingborg	22 (0.6)	90 (0.6)		
Huddinge	28 (0.8)	131 (0.9)		
Jönköping	49 (1.4)	679 (4.6)		
Kalmar	84 (2.4)	566 (3.8)		
Karlskrona	165 (4.8)	619 (4.2)		
Karlstad	65 (1.9)	778 (5.3)		
Karolinska Solna	255 (7.4)	1099 (7.4)		
Kristianstad	4 (0.1)	144 (1.0)		
Linköping	251 (7.3)	713 (4.8)		
Lund	785 (22.7)	1929 (13.1)		
Malmö	32 (0.9)	199 (1.3)		
Sahlgrenska	164 (4.7)	1387 (9.4)		
Skövde	50 (1.4)	487 (3.3)		
St Görans	42 (1.2)	62 (0.4)		
Sunderbyn	19 (0.5)	281 (1.9)		
Sundsvall	26 (0.8)	214 (1.4)		
SÖS	170 (4.9)	260 (1.8)		
Trollhättan	65 (1.9)	328 (2.2)		
Umeå	33 (1.0)	327 (2.2)		
Uppsala	160 (4.6)	810 (5.5)		
Västerås	85 (2.5)	283 (1.9)		
Örebro	125 (3.6)	998 (6.8)		
Östersund	6 (0.2)	47 (0.3)		
Östra sjukhuset	4 (0.1)	14 (0.1)		
**Stenosis class (%)**			0.213	0.049
A	168 (4.9)	928 (6.3)		
B1	882 (25.5)	4550 (30.8)		
B2	1346 (38.9)	6035 (40.9)		

	Thrombus aspiration	No thrombus aspiration	unweighted SMD	IP weighted SMD
C	1060 (30.6)	3214 (21.8)		
Other	6 (0.2)	33 (0.2)		
**Stenosis in culprit artery (%)**			0.441	0.151
50-69%	34 (1.0)	198 (1.3)		
70-89%	118 (3.4)	1081 (7.3)		
90-99%	510 (14.7)	4406 (29.9)		
100%	2800 (80.9)	9075 (61.5)		
**Angiography finding (%)**			0.164	0.032
Normal	2 (0.1)	16 (0.1)		
1 vessel	1957 (56.5)	7260 (49.2)		
2 vessels	931 (26.9)	4271 (28.9)		
3 vessels	450 (13.0)	2537 (17.2)		
Left main	117 (3.4)	659 (4.5)		
Missing	5 (0.1)	17 (0.1)		
**BMI (kg/m^2) (median [IQR])**	26.0 [24.0, 29.0]	26.0 [24.0, 29.0]	0.014	0.005
Missing (%)	839 (24.2)	3642 (24.7)		
**Smoking status (%)**			0.104	0.021
Never	1157 (33.4)	5596 (37.9)		
Ex smoker (> 1 month)	957 (27.6)	4045 (27.4)		
Current smoker	1038 (30.0)	3999 (27.1)		
Missing	310 (9.0)	1120 (7.6)		
**Diabetes (%)**	428 (12.4)	2292 (15.5)	0.091	0.024
**Hyperlipidemia treatment (%)**	724 (20.9)	3327 (22.5)	0.039	0.003
**Hypertension treatment (%)**	1340 (38.7)	6628 (44.9)	0.126	0.022
**Previous myocardial infarction (%)**	426 (12.3)	1996 (13.5)	0.036	0.016
**Previous percutaneous coronary intervention (%)**	368 (10.6)	1563 (10.6)	0.001	0.017
**Previous coronary artery bypass grafting (%)**	64 (1.8)	337 (2.3)	0.031	0.005
**Thrombolysis (%)**	16 (0.5)	54 (0.4)	0.015	0.007
**Warfarin (%)**	72 (2.1)	303 (2.1)	0.002	0.004
**Aspirin (%)**	3341 (96.5)	14347 (97.2)	0.040	0.009
**Clopidogrel or ticlopidine (%)**	2141 (61.8)	6357 (43.1)	0.383	0.031
**Prasugrel (%)**	118 (3.4)	623 (4.2)	0.042	0.028
**Heparin (%)**	2796 (80.8)	12648 (85.7)	0.132	0.018
**Low-molecular weight heparin (%)**	311 (9.0)	883 (6.0)	0.114	0.022
**Bivalirudin (%)**	1729 (49.9)	7081 (48.0)	0.039	0.021
**Glycoprotein IIb/IIIa inhibitors (%)**	1457 (42.1)	3906 (26.5)	0.334	0.076
**Heart rate (median [IQR])**	74.0 [61.0, 87.0]	75.0 [63.0, 88.0]	0.090	0.042
Missing (%)	245 (7.1)	784 (5.3)		
**Systolic blood pressure (median [IQR])**	138.0 [120.0, 157.0]	141.0 [125.0, 160.0]	0.181	0.066
Missing (%)	257 (7.4)	837 (5.7)		
**Diastolic blood pressure (median [IQR])**	80.0 [70.0, 95.0]	84.0 [72.0, 96.0]	0.088	0.033
Missing (%)	457 (13.2)	1475 (10.0)		

Web Table 5 - Baseline characteristics of eligible individuals for an observational emulation of a target trial of thrombus aspiration vs. no thrombus aspiration, SWEDEHEART registry, stratified into 2007-2010 & 2013-2016

	Before TASTE (Sep 07 to Jun 10)		After TASTE (Mar 13 to Jan 16)	
	**Thrombus aspiration**	**No thrombus aspiration**	**Thrombus aspiration**	**No thrombus aspiration**
**n**	2057	5602	1405	9158
**Age (yrs) (median [IQR])**	65.0 [58.0, 74.0]	68.0 [60.0, 77.0]	67.0 [57.0, 75.0]	68.0 [60.0, 77.0]
**Female (%)**	517 (25.1)	1712 (30.6)	370 (26.3)	2710 (29.6)
**Hospital (%)**				
Borås	8 (0.4)	55 (1.0)	7 (0.5)	88 (1.0)
Danderyd	33 (1.6)	46 (0.8)	101 (7.2)	317 (3.5)
Eskilstuna	3 (0.1)	4 (0.1)	64 (4.6)	394 (4.3)
Falun	181 (8.8)	256 (4.6)	30 (2.1)	382 (4.2)
Gävle	193 (9.4)	238 (4.2)	140 (10.0)	268 (2.9)
Halmstad	5 (0.2)	92 (1.6)	8 (0.6)	175 (1.9)
Helsingborg	1 (0.0)	0 (0.0)	21 (1.5)	90 (1.0)
Huddinge	8 (0.4)	49 (0.9)	20 (1.4)	82 (0.9)
Jönköping	10 (0.5)	214 (3.8)	39 (2.8)	465 (5.1)
Kalmar	71 (3.5)	299 (5.3)	13 (0.9)	267 (2.9)
Karlskrona	115 (5.6)	270 (4.8)	50 (3.6)	349 (3.8)
Karlstad	43 (2.1)	416 (7.4)	22 (1.6)	362 (4.0)
Karolinska Solna	133 (6.5)	503 (9.0)	122 (8.7)	596 (6.5)
Kristianstad	0 (0.0)	74 (1.3)	4 (0.3)	70 (0.8)
Linköping	119 (5.8)	256 (4.6)	132 (9.4)	457 (5.0)
Lund	596 (29.0)	708 (12.6)	189 (13.5)	1221 (13.3)
Malmö	29 (1.4)	173 (3.1)	3 (0.2)	26 (0.3)
Sahlgrenska	74 (3.6)	393 (7.0)	90 (6.4)	994 (10.9)
Skövde	5 (0.2)	188 (3.4)	45 (3.2)	299 (3.3)
St Görans	18 (0.9)	10 (0.2)	24 (1.7)	52 (0.6)
Sunderbyn	3 (0.1)	7 (0.1)	16 (1.1)	274 (3.0)
Sundsvall	11 (0.5)	44 (0.8)	15 (1.1)	170 (1.9)
SÖS	71 (3.5)	17 (0.3)	99 (7.0)	243 (2.7)
Trollhättan	57 (2.8)	166 (3.0)	8 (0.6)	162 (1.8)
Umeå	20 (1.0)	70 (1.2)	13 (0.9)	257 (2.8)
Uppsala	100 (4.9)	435 (7.8)	60 (4.3)	375 (4.1)
Västerås	55 (2.7)	25 (0.4)	30 (2.1)	258 (2.8)
Örebro	95 (4.6)	592 (10.6)	30 (2.1)	406 (4.4)
Östersund	0 (0.0)	0 (0.0)	6 (0.4)	47 (0.5)
Östra sjukhuset	0 (0.0)	2 (0.0)	4 (0.3)	12 (0.1)
**Stenosis class (%)**				
A	113 (5.5)	373 (6.7)	55 (3.9)	555 (6.1)
B1	492 (23.9)	1670 (29.8)	390 (27.8)	2880 (31.4)

	Before TASTE (Sep 07 to Jun 10)		After TASTE (Mar 13 to Jan 16)	
	**Thrombus aspiration**	**No thrombus aspiration**	**Thrombus aspiration**	**No thrombus aspiration**
B2	775 (37.7)	2220 (39.6)	571 (40.6)	3815 (41.7)
C	675 (32.8)	1314 (23.5)	385 (27.4)	1900 (20.7)
Other	2 (0.1)	25 (0.4)	4 (0.3)	8 (0.1)
**Stenosis in culprit artery (%)**				
50-69%	19 (0.9)	58 (1.0)	15 (1.1)	140 (1.5)
70-89%	62 (3.0)	376 (6.7)	56 (4.0)	705 (7.7)
90-99%	318 (15.5)	1661 (29.7)	192 (13.7)	2745 (30.0)
100%	1658 (80.6)	3507 (62.6)	1142 (81.3)	5568 (60.8)
**Angiography finding (%)**				
Normal	2 (0.1)	13 (0.2)	0 (0.0)	3 (0.0)
1 vessel	1134 (55.1)	2593 (46.3)	823 (58.6)	4667 (51.0)
2 vessels	543 (26.4)	1728 (30.8)	388 (27.6)	2543 (27.8)
3 vessels	307 (14.9)	1007 (18.0)	143 (10.2)	1530 (16.7)
Left main	70 (3.4)	250 (4.5)	47 (3.3)	409 (4.5)
Missing	1 (0.0)	11 (0.2)	4 (0.3)	6 (0.1)
**BMI (kg/m^2) (median [IQR])**	26.0 [24.0, 29.0]	26.0 [24.0, 29.0]	27.0 [24.0, 29.0]	26.0 [24.0, 29.0]
Missing (%)	480 (23.3)	1567 (28.0)	359 (25.6)	2075 (22.7)
**Smoking status (%)**				
Never	664 (32.3)	2121 (37.9)	493 (35.1)	3475 (37.9)
Ex smoker (> 1 month)	563 (27.4)	1440 (25.7)	394 (28.0)	2605 (28.4)
Current smoker	641 (31.2)	1579 (28.2)	397 (28.3)	2420 (26.4)
Missing	189 (9.2)	462 (8.2)	121 (8.6)	658 (7.2)
**Diabetes (%)**	251 (12.2)	802 (14.3)	177 (12.6)	1490 (16.3)
**Hyperlipidemia treatment (%)**	404 (19.6)	1195 (21.3)	320 (22.8)	2132 (23.3)
**Hypertension treatment (%)**	777 (37.8)	2296 (41.0)	563 (40.1)	4332 (47.3)
**Previous myocardial infarction (%)**	256 (12.4)	811 (14.5)	170 (12.1)	1185 (12.9)
**Previous percutaneous coronary intervention (%)**	223 (10.8)	609 (10.9)	145 (10.3)	954 (10.4)
**Previous coronary artery bypass grafting (%)**	31 (1.5)	133 (2.4)	33 (2.3)	204 (2.2)
**Thrombolysis (%)**	10 (0.5)	19 (0.3)	6 (0.4)	35 (0.4)
**Warfarin (%)**	30 (1.5)	106 (1.9)	42 (3.0)	197 (2.2)
**Aspirin (%)**	1980 (96.3)	5391 (96.2)	1361 (96.9)	8956 (97.8)
**Clopidogrel or ticlopidine (%)**	1903 (92.5)	5247 (93.7)	238 (16.9)	1110 (12.1)
**Prasugrel (%)**	75 (3.6)	49 (0.9)	43 (3.1)	574 (6.3)
**Heparin (%)**	1506 (73.2)	4027 (71.9)	1290 (91.8)	8621 (94.1)
**Low-molecular weight heparin (%)**	281 (13.7)	781 (13.9)	30 (2.1)	102 (1.1)
**Bivalirudin (%)**	836 (40.6)	1790 (32.0)	893 (63.6)	5291 (57.8)
**Glycoprotein IIb/IIIa inhibitors (%)**	1119 (54.4)	2942 (52.5)	338 (24.1)	964 (10.5)
**Heart rate (median [IQR])**	72.0 [60.0, 85.0]	74.0 [62.0, 87.0]	75.0 [62.0, 88.0]	75.0 [64.0, 89.0]
Missing (%)	216 (10.5)	595 (10.6)	29 (2.1)	189 (2.1)
**Systolic blood pressure (median [IQR])**	138.0 [120.0, 156.0]	140.0 [122.0, 160.0]	139.5 [120.0, 158.0]	144.0 [125.0, 162.2]
Missing (%)	226 (11.0)	647 (11.5)	31 (2.2)	190 (2.1)

	Before TASTE (Sep 07 to Jun 10)		After TASTE (Mar 13 to Jan 16)	
	**Thrombus aspiration**	**No thrombus aspiration**	**Thrombus aspiration**	**No thrombus aspiration**
**Diastolic blood pressure (median [IQR])**	80.0 [70.0, 95.0]	80.0 [70.0, 95.0]	82.5 [70.0, 95.0]	85.0 [74.0, 97.0]
Missing (%)	316 (15.4)	803 (14.3)	141 (10.0)	672 (7.3)

Web Table 6 - Estimated 1-year risk, risk difference, and risk ratios from an observational emulation of a target trial of thrombus aspiration vs. no thrombus aspiration, SWEDEHEART registry, stratified into 2007-2010 & 2013-2016

		Risk (%, 95% CI)			
**Outcome**	**Model**	**Thrombus aspiration**	**No thrombus aspiration**	**Risk difference (%, 95% CI)**	**Risk ratio (95% CI)**
**Before TASTE**					
Death	Age and sex adjusted	7.7 (6.1, 9.3)	7.3 (6.3, 8.3)	0.4 (-1.4, 2.2)	1.05 (0.89, 1.25)
	Full adjustment*	7.3 (5.8, 8.8)	7.5 (6.5, 8.4)	-0.2 (-2.0, 1.6)	0.97 (0.81, 1.17)
Myocardial infarction	Age and sex adjusted	4.2 (3.0, 5.4)	5.1 (4.3, 5.9)	-0.9 (-2.3, 0.6)	0.83 (0.66, 1.03)
	Full adjustment*	4.3 (3.1, 5.6)	5.0 (4.3, 5.8)	-0.7 (-2.3, 0.8)	0.85 (0.68, 1.08)
**After TASTE**					
Death	Age and sex adjusted	9.4 (7.4, 11.4)	7.2 (6.4, 8.0)	2.2 (0.0, 4.4)	1.31 (1.10, 1.56)
	Full adjustment*	8.5 (6.7, 10.4)	7.3 (6.6, 8.1)	1.2 (-0.7, 3.2)	1.17 (0.98, 1.39)
Myocardial infarction	Age and sex adjusted	3.8 (2.4, 5.2)	3.4 (2.9, 3.9)	0.4 (-1.0, 1.9)	1.12 (0.86, 1.47)
	Full adjustment*	3.6 (2.2, 5.1)	3.4 (2.9, 3.9)	0.2 (-1.3, 1.8)	1.06 (0.79, 1.44)

*Adjusted at baseline for: age, sex, hospital, diabetes, body mass index, smoking, hyperlipidemia, hypertension, previous infarction, previous percutaneous coronary intervention, previous coronary artery bypass graft, stenosis class, proportion stenosis, angiography finding, heart rate, systolic blood pressure, diastolic blood pressure, thrombolysis, warfarin, aspirin, clopidogrel, prasugrel, heparin, low molecular weight heparin, bivalirudin, and Gp2b3a inhibitors

Web Table 7 - Sensitivity analysis - Estimated 1-year risk, risk difference, and risk ratios from an observational emulation of a target trial of thrombus aspiration vs. no thrombus aspiration, SWEDEHEART register, 2007-2016, without application of the 50% minimum stenosis eligibility criterion

	Risk (%, 95% CI)*			
**Outcome**	**Thrombus aspiration**	**No thrombus aspiration**	**Risk difference (%, 95% CI)***	**Risk ratio (95% CI)***
Death	8.1 (7.0, 9.3)	7.3 (6.8, 7.9)	0.8 (-0.5, 2.0)	1.11 (0.98, 1.24)
Myocardial infarction	4.0 (3.1, 4.9)	4.3 (3.9, 4.8)	-0.3 (-1.3, 0.7)	0.94 (0.79, 1.11)

*Adjusted at baseline for: age, sex, hospital, diabetes, body mass index, smoking, hyperlipidemia, hypertension, previous infarction, previous percutaneous coronary intervention, previous coronary artery bypass graft, stenosis class, proportion stenosis, angiography finding, heart rate, systolic blood pressure, diastolic blood pressure, thrombolysis, warfarin, aspirin, clopidogrel, prasugrel, heparin, low molecular weight heparin, bivalirudin, and Gp2b3a inhibitors

Web Table 8 - Sensitivity analysis - Estimated 1-year risk, risk difference, and risk ratios from an observational emulation of a target trial of thrombus aspiration vs. no thrombus aspiration, SWEDEHEART register, 2007-2016, adjusted for age and sex

	Risk (%, 95% CI)			
**Outcome**	**Thrombus aspiration***	**No thrombus aspiration***	**Risk difference (%, 95% CI)***	**Risk ratio (95% CI)***
Death	8.5 (7.2, 9.8)	7.2 (6.6, 7.8)	1.3 (-0.1, 2.7)	1.18 (1.04, 1.34)
Myocardial infarction	4.1 (3.0, 5.1)	4.0 (3.6, 4.5)	0.0 (-1.1, 1.2)	1.01 (0.83, 1.22)

*Adjusted at baseline for: age and sex

Web Table 9 - Sensitivity analysis - Difference in risk differences between age and sex adjusted, and fully adjusted models from an observational emulation of a target trial of thrombus aspiration vs. no thrombus aspiration, SWEDEHEART register, 2007-2016

**Outcome**	**Difference in risk difference**
Death	-0.6 (-1.4, 0.1)
Myocardial infarction	-0.2 (-0.6, 0.2)

Web Table 10 - Sensitivity analysis - Estimated 1-year risk, risk difference, and risk ratios from an observational emulation of a target trial of thrombus aspiration vs. no thrombus aspiration, SWEDEHEART register, 2007-2016, with individuals censored at death

	Risk (%, 95% CI)*			
**Outcome**	**Thrombus aspiration**	**No thrombus aspiration**	**Risk difference (%, 95% CI)***	**Risk ratio (95% CI)***
Myocardial infarction	4.1 (3.2,5.1)	4.3 (3.8,4.8)	-0.2 (-1.2,0.8)	0.95 (0.80,1.14)

*Adjusted at baseline for: age, sex, hospital, diabetes, body mass index, smoking, hyperlipidemia, hypertension, previous infarction, previous percutaneous coronary intervention, previous coronary artery bypass graft, stenosis class, proportion stenosis, angiography finding, heart rate, systolic blood pressure, diastolic blood pressure, thrombolysis, warfarin, aspirin, clopidogrel, prasugrel, heparin, low molecular weight heparin, bivalirudin, and Gp2b3a inhibitors

Web Table 11 - Sensitivity analysis - Estimated 1-year risk, risk difference, and risk ratios from an observational emulation of a target trial of thrombus aspiration vs. no thrombus aspiration, SWEDEHEART register, 2007-2016, with a complete case analysis

	Risk (%, 95% CI)*			
**Outcome**	**Thrombus aspiration**	**No thrombus aspiration**	**Risk difference (%, 95% CI)***	**Risk ratio (95% CI)***
Death	6.5 (4.7,8.3)	5.6 (4.7,6.4)	0.9 (-1.1,2.9)	1.16 (0.92,1.46)
Myocardial infarction	4.2 (2.7,5.7)	4.4 (3.5,5.2)	-0.2 (-2.0,1.6)	0.96 (0.71,1.28)

*Adjusted at baseline for: age, sex, hospital, diabetes, body mass index, smoking, hyperlipidemia, hypertension, previous infarction, previous percutaneous coronary intervention, previous coronary artery bypass graft, stenosis class, proportion stenosis, angiography finding, heart rate, systolic blood pressure, diastolic blood pressure, thrombolysis, warfarin, aspirin, clopidogrel, prasugrel, heparin, low molecular weight heparin, bivalirudin, and Gp2b3a inhibitors

Web Table 12 - Sensitivity analysis - Estimated 1-year risk, risk difference, and risk ratios from an observational emulation of a target trial of thrombus aspiration vs. no thrombus aspiration, SWEDEHEART register, 2007-2016, with alternative myocardial infarction definition

	Risk (%, 95% CI)*			
**Outcome**	**Thrombus aspiration**	**No thrombus aspiration**	**Risk difference (%, 95% CI)***	**Risk ratio (95% CI)***
Myocardial infarction	3.8 (2.8, 4.8)	3.8 (3.4, 4.3)	0.0 (-1.2, 1.1)	0.99 (0.81, 1.21)

*Adjusted at baseline for: age, sex, hospital, diabetes, body mass index, smoking, hyperlipidemia, hypertension, previous infarction, previous percutaneous coronary intervention, previous coronary artery bypass graft, stenosis class, proportion stenosis, angiography finding, heart rate, systolic blood pressure, diastolic blood pressure, thrombolysis, warfarin, aspirin, clopidogrel, prasugrel, heparin, low molecular weight heparin, bivalirudin, and Gp2b3a inhibitors

Web Table 13 - Sensitivity analysis - Estimated 1-year risk, risk difference, and risk ratios from an observational emulation of a target trial of thrombus aspiration vs. no thrombus aspiration, SWEDEHEART register, 2013-2016, additionally adjusted for Killip class

	Risk (%, 95% CI)*			
**Outcome**	**Thrombus aspiration**	**No thrombus aspiration**	**Risk difference (%, 95% CI)***	**Risk ratio (95% CI)***
Death	8.4 (6.6, 10.1)	7.3 (6.6, 8.1)	1.0 (-0.9, 2.9)	1.14 (0.96, 1.35)
Myocardial infarction	3.6 (2.3, 5.0)	3.4 (2.9, 3.9)	0.2 (-1.2, 1.7)	1.06 (0.80, 1.40)

*Adjusted at baseline for: killip class, age, sex, hospital, diabetes, body mass index, smoking, hyperlipidemia, hypertension, previous infarction, previous percutaneous coronary intervention, previous coronary artery bypass graft, stenosis class, proportion stenosis, angiography finding, heart rate, systolic blood pressure, diastolic blood pressure, thrombolysis, warfarin, aspirin, clopidogrel, prasugrel, heparin, low molecular weight heparin, bivalirudin, and Gp2b3a inhibitors

Web Table 14 - Sensitivity analysis - Estimated 1-year risk, risk difference, and risk ratios from an observational emulation of a target trial of thrombus aspiration vs. no thrombus aspiration, SWEDEHEART register, 2013-2016, additionally adjusted for time period (before or after TASTE)

	Risk (%, 95% CI)*			
**Outcome**	**Thrombus aspiration**	**No thrombus aspiration**	**Risk difference (%, 95% CI)***	**Risk ratio (95% CI)***
Death	8.0 (6.7,9.3)	7.3 (6.8,7.9)	0.7 (-0.7,2.1)	1.09 (0.96,1.24)
Myocardial infarction	3.9 (2.9,4.9)	4.1 (3.6,4.5)	-0.2 (-1.3,0.9)	0.95 (0.78,1.16)

*Adjusted at baseline for: period, age, sex, hospital, diabetes, body mass index, smoking, hyperlipidemia, hypertension, previous infarction, previous percutaneous coronary intervention, previous coronary artery bypass graft, stenosis class, proportion stenosis, angiography finding, heart rate, systolic blood pressure, diastolic blood pressure, thrombolysis, warfarin, aspirin, clopidogrel, prasugrel, heparin, low molecular weight heparin, bivalirudin, and Gp2b3a inhibitors

Web Table 15 - Sensitivity analysis - Estimated 1-year risks from an observational emulation of a target trial for thrombus aspiration and no thrombus aspiration, SWEDEHEART register, 2013-2016, when data are stratified by exposure ad outcome models are fit in each arm

	Risk (%, 95% CI)*	
**Outcome**	**Thrombus aspiration**	**No thrombus aspiration**
Death	7.5 (6.9,8.0)	7.5 (6.3,8.6)
Myocardial infarction	4.1 (3.6,4.5)	4.0 (3.0,4.9)

*Adjusted at baseline for: age, sex, hospital, diabetes, body mass index, smoking, hyperlipidemia, hypertension, previous infarction, previous percutaneous coronary intervention, previous coronary artery bypass graft, stenosis class, proportion stenosis, angiography finding, heart rate, systolic blood pressure, diastolic blood pressure, thrombolysis, warfarin, aspirin, clopidogrel, prasugrel, heparin, low molecular weight heparin, bivalirudin, and Gp2b3a inhibitors

Web Table 16 - Sensitivity analysis - Estimated 1-year risk, risk difference, and risk ratios from an observational emulation of a target trial of thrombus aspiration vs. no thrombus aspiration, SWEDEHEART register, 2013-2016, adjusted for baseline confounding using inverse probability weighting

	**Risk (%, 95% CI)***			
**Outcome**	**Thrombus aspiration**	**No thrombus aspiration**	**Risk difference (%, 95% CI)***	**Risk ratio (95% CI)***
Death	8.3 (7.3,9.2)	7.4 (7.1,7.7)	0.9 (-0.1,1.9)	1.12 (0.99,1.26)
Myocardial infarction	3.7 (3.1,4.3)	4.1 (3.9,4.3)	-0.4 (-1.1,0.2)	0.90 (0.75,1.07)

*Adjusted at baseline for: period, age, sex, hospital, diabetes, body mass index, smoking, hyperlipidemia, hypertension, previous infarction, previous percutaneous coronary intervention, previous coronary artery bypass graft, stenosis class, proportion stenosis, angiography finding, heart rate, systolic blood pressure, diastolic blood pressure, thrombolysis, warfarin, aspirin, clopidogrel, prasugrel, heparin, low molecular weight heparin, bivalirudin, and Gp2b3a inhibitors

Web Figure 1 - Average 1-year hazard ratio from TASTE and estimated 1-year risk ratios from an observational emulation of a target trial of thrombus aspiration vs. no thrombus aspiration, SWEDEHEART registry, stratified into 2007-2010 & 2013-2016

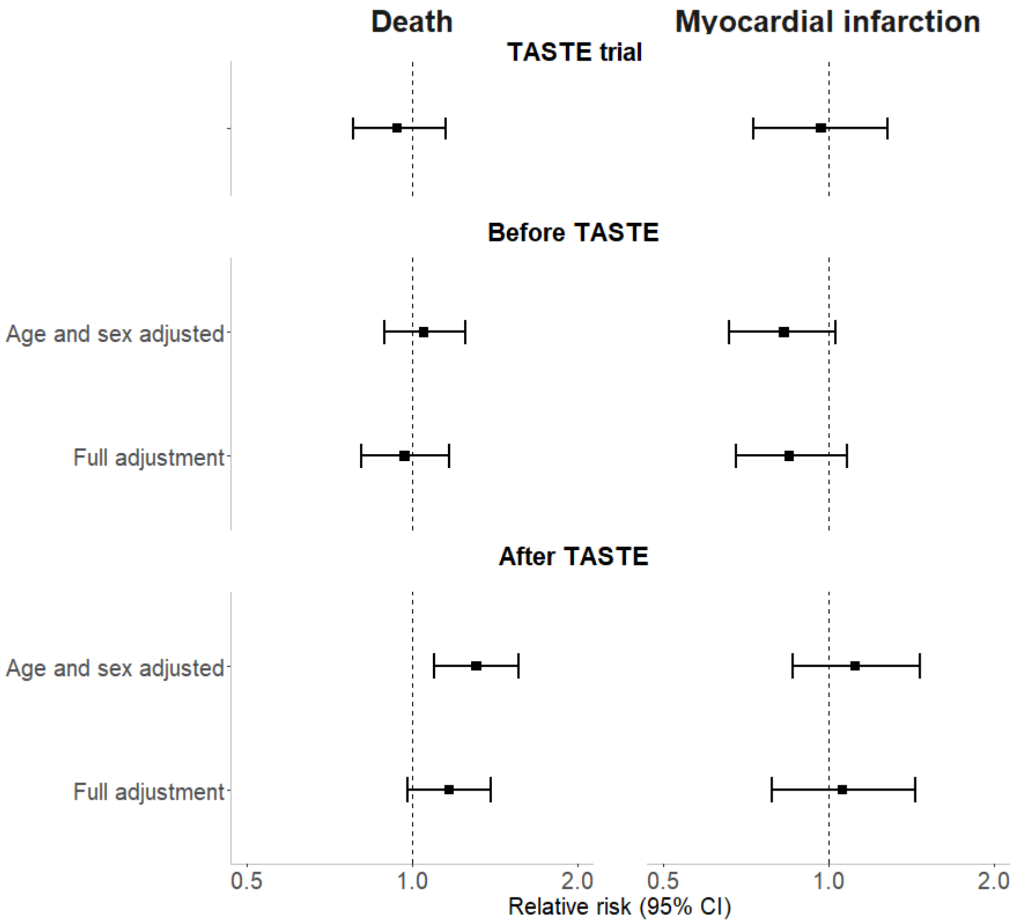

Supplement: Web_Material_kwac098 [file web_material_kwac098.pdf]
